# A Randomized, Double-Blind, Placebo-Controlled Trial to Assess the Efficacy and Safety of *Lactiplantibacillus plantarum* CJLP243 in Patients with Functional Diarrhea and High Fecal Calprotectin Levels

**DOI:** 10.3390/nu14020389

**Published:** 2022-01-17

**Authors:** MinAh Jung, Susie Jung, NamKyu Kim, HeeYoon Ahn, HyunSun Yun, Kyu-Nam Kim

**Affiliations:** 1 Department of Family Practice and Community Health, Ajou University School of Medicine, 164 World cup-ro, Yeongtong-gu, Suwon 16499, Korea; zpcus0512@naver.com (M.J.); lovehrh@naver.com (S.J.); childrenrock@hanmail.net (N.K.); 2 Health R&D Center, CJ CheilJedang Corporation, Suwon 16495, Korea; hy.ahn@cj.net (H.A.); hs.yun@cj.net (H.Y.)

**Keywords:** functional diarrhea, gut microbiota, *Lactiplantibacillus plantarum*, fecal calprotectin

## Abstract

Micro-inflammation in the gut, assessed by fecal calprotectin (FC), is considered a component of the pathogenesis of functional diarrhea (FD). Since probiotics may suppress micro-inflammation in the intestine by competing with harmful bacteria, we hypothesized that they would reduce the ratio of loose stool symptoms and gut inflammation in patients with FD. We conducted a double-blind, placebo-controlled trial to assess the clinical and laboratory effects of *Lactobacillus plantarum* CJLP243 in FD patients with elevated FC levels for two months. Twenty-four patients diagnosed with FD with elevated FC levels were randomly assigned to either a probiotic group or a placebo group. After 2 months, 10 patients in the probiotic group and 12 patients in the placebo group completed the study, and FD symptoms, FC values, and intestinal flora were re-evaluated in these subjects. The percentage of subjects who had adequate FD relief (decrease in loose stool frequency) in the probiotic group was significantly increased after two months compared with the baseline. In addition, the probiotic group showed a statistically significant decrease in log-transformed FC values compared with the pre-treatment group, whereas the placebo group showed no difference before and after the intervention. Furthermore, the levels of *Leuconostoc* genus organisms in the gut microbiota composition in the probiotic group increased significantly after the end of the study compared with the baseline values. In this preliminary exploratory research, we found that two months of *Lactiplantibacillus plantarum* CJLP243 treatment resulted in FD symptom improvement, reduced FC values, and increased *Leuconostoc* levels, suggesting that the intake of *Lactiplantibacillus plantarum* was helpful in those patients. These findings need to be validated via further clinical studies.

## 1. Introduction

Functional diarrhea (FD) is characterized by chronic or recurrent diarrhea that is not explained by structural or biochemical abnormalities and is distinguished from diarrhea-predominant irritable bowel syndrome (IBS), where abdominal pain is the main symptom [1]. Recent results from the Rome Foundation Global Study showed that, according to the Rome IV criteria, FD had a prevalence of about 4.7%, which was higher than the prevalence of IBS (4.1%), in which abdominal pain is the main symptom [2]. In addition, FD has been associated with major impacts on the quality of life due to loose or watery stool frequency, urgency, incontinence, psychiatric conditions, and chronic fatigue [3]. Despite its high prevalence and comorbidities, the treatments primarily target patient’s symptoms rather than the underlying etiologic mechanisms, as the pathophysiologic basis of FD is poorly understood. However, recent studies reported that microscopic colitis (MC) may play a role in the pathogenesis of FD. Indeed, a recently published systematic review with meta-analysis reported that MC and FD shared overlapping symptoms, and the prevalence of MC among patients with suspected FD was about 9% [4]. Previous studies have reported the presence of inflammation in biopsy samples of the intestinal mucosa of patients with FD compared with controls [5,6]. Taken together, these findings suggest that inflammation of the gut plays a role in FD. One of the most commonly used markers of gut inflammation in clinical practice is fecal calprotectin (FC). Calprotectin is a cytosolic protein complex released from neutrophilic granulocytes, monocytes, and macrophages in the gut during intestinal inflammation [7]. Thus, FC has been used as a biomarker for colonic inflammation.

Probiotics are defined as live microorganisms that bring beneficial effects to intestinal mucosa when consumed in suitable amounts [8]. Probiotics are thought to metabolically compete with pathogens to restore the intestinal mucosal barrier and reduce gut inflammation [9]. In particular, *Lactiplantibacillus plantarum* was reported to relieve diarrhea symptoms by regulating intestinal inflammation in a recent animal study [10]. Therefore, we hypothesized that the oral administration of *L. platarum* could reduce loose stool frequency and intestinal inflammation in patients with FD by restoring intestinal microbial balance. The study’s primary aim was to assess the frequency of loose or watery stools and the changes in FC values in FD patients with elevated FC levels after the administration of *L. plantarum* CJLP243 for two months compared with placebo. The secondary aim was to assess the effect on fecal microbiota and the safety of *L. plantarum* CJLP243.

## 2. Materials and Methods

### 2.1. Study Population and Design

**Figure 1:**
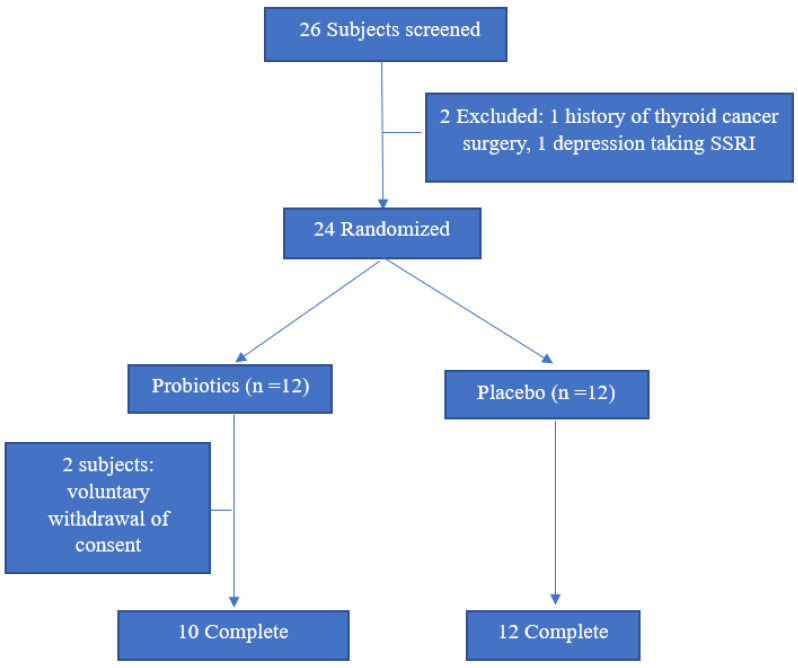
Flow chart outlining the current study protocol.

From August 2010 to July 2021, subjects with FD according to Rome IV criteria were recruited from the Ajou University Health Promotion Center and Department of Family Medicine through detailed interviews during general medical examinations or regular visits. The Rome IV criteria defines FD as a frequency loose or watery stools of at least 25% of the time devoid of IBS symptoms in the past 3 months, and where abdominal pain or bloating was not the predominant symptom. The stool status of the subjects was obtained through detailed interviews with the extent to which stools corresponded to 6 or 7 on the Bristol scale during the last 3 months at the time of hospital visit. Medical record was reviewed for any organic gastrointestinal diseases on gastroduodenoscopy or colonoscopy within the last one or two years, respectively. Subjects with FD were excluded from the study if they had any history of the following: (i) gastric ulcer or inflammatory bowel diseases; (ii) surgery manipulating the intestines (except appendectomy); (iii) chronic renal failure, liver cirrhosis, congestive heart failure, or thyroid disease; (iv) prescription for motility drugs; or (v) any malignancy. The subjects were tested for FC to confirm microscopic intestinal inflammation and a total of 26 patients with an FC score of 11.5 or higher were enrolled. After a detailed medical record review, two of them were excluded due to thyroid cancer surgery and administration of selective serotonin reuptake inhibitor that affects bowel movement. Finally, a total of 24 subjects participated in this study, and 12 each were assigned either to the probiotic or the placebo group, using block randomization (Figure 1). The subjects were not permitted to take antibiotics, proton pump inhibitor, other probiotics, or intestinal motility drugs during the study period. Smoking status was classified as current smoker or non-smoker, depending on their habit at the time of enrollment. Hypertension and dyslipidemia were checked out through medical record or interview. Alcohol consumption was assessed in grams of ethanol consumed per week using the graduated frequency method [11].

*L. plantarum* CJLP243 (KCCM 11045P) was manufactured by the Health R&D Center, CJ CheilJedang Corporation (Suwon, Korea). *L. plantarum* CJLP243 is a lactic acid bacterium isolated from Kimchi and has been shown to exhibit immune-stimulating activity through the enhancement of T cell activation in BALB/c mice [12]. *L. plantarum* CJLP243 at a dosage of 1.0 × 10^10^ colony-forming units (CFU) in 2 g of powder once a day was mixed with maltodextrin and glucose anhydrocrystalline and stored at 4 °C until administration in airtight alu-bags. Placebo was composed of maltodextrin and glucose anhydrocrystalline. Final products had identical shape, texture, and appearance. The subjects in each group received a two month supply of the probiotic or placebo.

All patients signed an informed consent form prior to enrollment in the study. The study was conducted in accordance with the Declaration of Helsinki, and the protocol was approved by the Ethics Committee of Ajou University Hospital (approval no.: AJIRB-MED-FOD-20-179). The clinical trial number was acquired from the Clinical Research Information Service (CRIS registration number: KCT0006776).

### 2.2. Measurements

#### 2.2.1. Definition of Adequate Relief of FD Symptoms

Since there are no guidelines to define the improvement of FD, we employed the Food and Drug Administration’s (FDA’s) recommendations that defined adequate relief in diarrhea-predominant IBS [13]. Thus, we delineated adequate relief of FD symptoms as cases in which the percentage corresponding to type 6 or 7 on the Bristol stool scale, decreased by more than 30% after 2 months, compared with the baseline.

#### 2.2.2. FC and Fecal Microbiology Assay

Analysis of FC and fecal microbiology was performed at the time of study enrollment and at the end of the study. FC analysis was performed at the Institute of Applied Technology for Green Cross LabCell (Yongin, Korea) and fecal flora analysis was performed at the GC Genome and Corp (Yongin, Korea). Calprotectin level was measured using an ImmunoCAP 250^®^ (Aloka, Japan) with a calprotectin fluorescence enzyme immunoassay (FEIA) kit (Phadia AB, Sweden). The FC level is expressed as mg/kg feces. FC levels less than 11.5 mg/kg could not be measured, as shown in our previous study [14], so a value greater than 11.5 mg/kg was defined as high fecal calprotectin. The microbiome was analyzed using next-generation sequencing methods. In the classified taxonomy, the features included in mitochondria, chloroplasts, and archaea were filtered. The average number of unassigned features among taxonomy at the genus level was about 3.166%. Alpha diversity sampled 1000 reads and Shannon’s index was calculated.

### 2.3. Sample Size Estimation

This study was conducted as a pilot study because there are no reliable data on probiotics in patients with FD with elevated FC levels. Initially, a total of 20 patients were planned, 10 in each group, and a total of 24 patients were enrolled in consideration of the dropout rate of 20% in each group.

### 2.4. Statistical Analysis

We compared and analyzed 10 participants in the probiotic group and 12 in the placebo group who completed the 2-month study. The distribution of FC values was right-skewed, and then the result of FC values was analyzed by natural log-transformation. The one-sample Kolmogorov–Smirnov test was performed to check whether the continuous variables showed a normal distribution, and it was significant, with a *p*-value of >0.05. Therefore, we compared the continuous variables between the groups at baseline and after two months using the independent *t*-test and a paired *t*-test. We also compared the categorical variables, including the sociodemographic variables, baseline clinical variables, and the proportion of patients with improvement of FD symptoms in both groups using Fisher’s exact test or the chi-squared test. In fecal microbiology analysis, linear discriminant analysis (LDA) effect size (LEfSe) analysis was performed to estimate the effect size of differentially abundant features with biological consistency and statistical significance. Herein, the *p*-value for the statistical test was set at 0.05, and the LDA score for discriminative features was set at more than 2.0.

## 3. Results

Table 1 shows the variables in the probiotic and the placebo group at baseline. There were no differences between the two groups in age, sex, body mass index, current smoking status, weekly alcohol consumption, hypertension, and dyslipidemia. There was no difference between the two groups in the rate of loose stools and the mean FC value.

**Figure 2:**
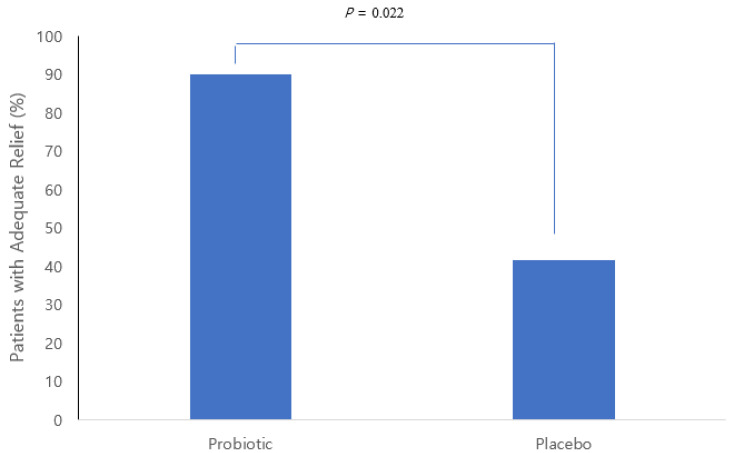
Percentage of patients with adequate FD relief at two months.

We defined adequate treatment relief in the FD patients when the ratio of loose stools decreased by 30% or more from the baseline. Figure 2 shows the percentage of subjects who had adequate FD relief from treatment in the probiotic group and the placebo group. The probiotic group showed an appropriate treatment response in 90% (9 of 10) and the placebo group in 41.7% (5 of 12), which showed a statistically significant difference between the 2 groups.

**Figure 3:**
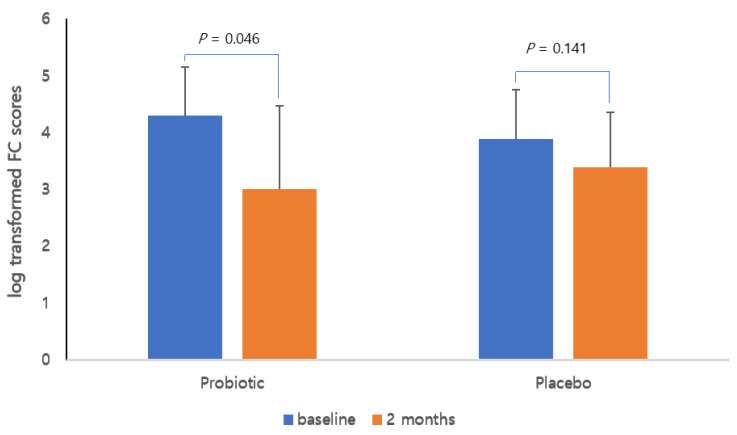
Changes in log-transformed fecal calprotectin levels in each group. Line graphs show the mean and standard deviation.

Figure 3 shows the changes in log-transformed FC values after two months of treatment compared with baseline in both groups. The probiotic group showed a statistically significant decrease in log-transformed FC values compared with the baseline, whereas the placebo group showed no difference before and after the treatment.

**Figure 4:**
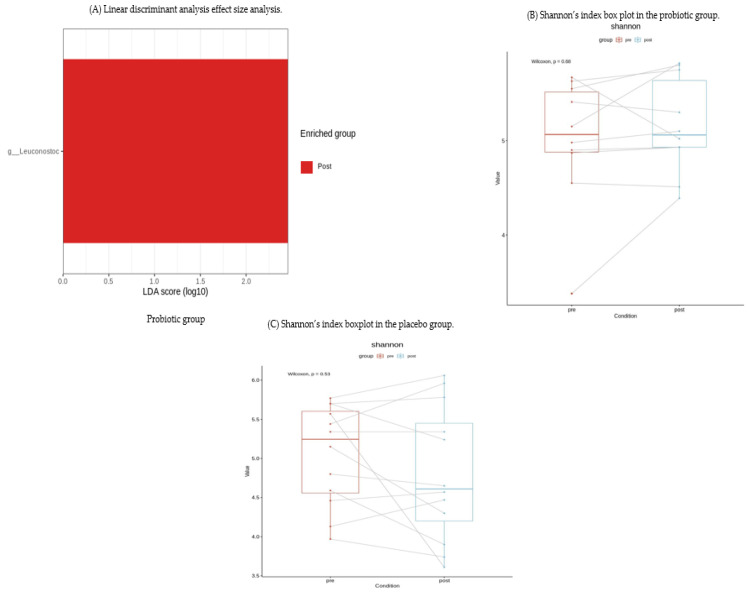
Effect of the probiotic on gut microbiota composition. (**A**) *Leuconostoc* genus organisms were significantly increased in the probiotic group only compared with before treatment (LDA = 2.458, *p* = 0.002). No statistically significant bacteria were found in the placebo group (data not shown). g_ Leuconostoc, genus Leuconostoc. (**B**) In the probiotic group, Shannon diversity showed no difference before and after treatment. (**C**) In the placebo group, Shannon diversity showed no difference before and after treatment.

We performed LEfSe analysis in both groups and found that the *Leuconostoc* genus organisms in the gut microbiota composition in the probiotic group increased significantly after two months compared with the baseline (*p* = 0.002) (Figure 4A). However, in the alpha-diversity analysis, there were no significant changes in either group after two months.

Table 2 shows the leukocyte, hemoglobin levels, liver function, and renal function test results at baseline and at the end of the study (two months) in the probiotic and placebo groups. There was no statistically significant difference in the variables measured at baseline and at the end of the study in either group.

## 4. Discussion

The present study was a double-blind randomized study to examine the effect of *L. plantarum*, a known beneficial probiotic, after two months of treatment in patients with FD and elevated FC values. We observed a statistically significant decrease in loose stools—which was the primary goal of this study—in the probiotic group compared with the placebo group after two months. In addition, FC, an indicator of colonic inflammation, was also decreased significantly after two months compared with baseline in the probiotic group. Furthermore, in the gut microbiota analysis investigating significant changes in both groups, *Leuconostoc* genus organisms in the gut microbiota increased from baseline in the probiotic group.

*L. plantarum* is Gram-positive lactic acid bacteria, and a probiotic strain belonging to the phylum Firmicutes and the genus *Lactiplantibacillus* [15]. This particular strain was identified after oral ingestion in biopsied samples of the jejunum and rectum, demonstrating that this strain was able to colonize the human intestinal mucosa [16,17]. Although there has been no study on the effect of *L. plantarum* in FD patients, previous studies investigated the effect of *L. plantarum* on diarrhea symptoms. Yue et al. [9] demonstrated that *L. plantarum* alleviated enterotoxigenic *Escherichia coli*-induced diarrhea by regulating inflammatory cytokines, rebalancing the gut microbiota, and modulating short-chain fatty acids generation. Similar results were obtained in a placebo-controlled study by Ducrotté et al. [18], where the overall reductions in stool frequency and frequency of feeling incomplete emptying were significantly greater in the *L. plantarum* group compared with the placebo group over a four-week period. Another study also examined the efficacy of *L. plantarum* in the treatment of recurrent *Clostridium difficile*-associated diarrhea [19]. This study reported that, although not statistically significant, there was a tendency toward fewer recurrences in the *Lactiplantibacillus* group (4 of 11) compared with the placebo group (6 of 9). In summary, the above-mentioned studies support that the administration of *L. plantarum* is useful in improving the symptoms of FD.

The exact mechanism by which *L. plantarum* improves symptoms in patients with FD is not yet known, but it may be partially explained by the following studies. First, exopolysaccharides from *L. plantarum* reduced the production of pro-inflammatory cytokines (tumor necrosis factor-α, interferon-gamma, and interleukin (IL)-12) and enhanced the anti-inflammatory cytokine IL-10 [20]. In addition, *L. plantarum* is a promising probiotic bacterium that can potentially alleviate oxidative stress [21]. These results are consistent with our findings that FC levels, indicating MC, were significantly decreased in the *L. plantarum* group. Second, supplementation with *L. plantarum* could restore unbalanced gut microbiota [10]. Cheng et al. [22] also demonstrated that when mice with gut dysbiosis developed by antibiotic administration ingested *L. plantarum*, the intestinal flora was changed, close to that in the control group. The present study also demonstrated that *L.plantarum* treatment significantly increased *Leuconostoc* genus organisms in the LEfSe analysis of meaningful changes in gut microbiota composition. *Leuconostoc* is a lactic acid-producing bacterium present in Kimchi that is commonly consumed by Koreans [23]. Since *Leuconostoc* is a lactic acid-producing bacteria like *L. plantarum*, it may have contributed to suppress the growth of some pathogenic bacteria and improved the immune system by lowering the acidity in the intestine [24]. Taken together, our finding suggests that the intake of *L. plantarum* caused an increase in *Leuconostoc* genus organisms, which might help to improve FD symptoms and reduce microscopic intestinal inflammation.

There were few drawbacks to the study design used in this study. The present study was conducted as a pilot study with a small sample size because no previous study has examined the therapeutic effect of *L. plantarum* in patients with FD. Thus, we did not use a formal sample size calculation and selected a size that was appropriate for addressing our study objectives. We found an increase in *Leuconostoc* organisms alone at the genus level in the intestinal flora analysis in the probiotic group, and the composition of fecal microbiota at the species level did not identify any significant strains in either group. No significant change was found in the probiotic group in the alpha diversity analysis. These findings may also be due to the small number of subjects, so a larger study with a sufficient number of subjects is needed in the future. Nonetheless, this study was the first randomized study assessing the effects of *L. plantarum* in FD patients with elevated FC values to show meaningful results in the FD subjects with high FC values.

In conclusion, the results of the first preliminary double-blind randomized controlled human study demonstrated that a daily intake of 10 billion *L. plantarum* CJLP243 bacteria for 60 days was effective in improving the symptoms of diarrhea and reducing FC levels compared with placebo in FD patients, according to the Rome IV criteria. Furthermore, *Leuconostoc* was increased at the genus level of intestinal bacteria. These findings support previous studies that showed therapeutic effects in patients with functional gastrointestinal symptoms, either alone or in combination with beneficial strains of probiotics [25,26]. Therefore, the results of this study are encouraging, and well-designed, large-scale future studies are necessary to support *L. plantarum*’s efficacy and safety, and to prove sustainability of the improvement.

## Figures and Tables

**Table 1:** Baseline characteristics of the study subjects.

	Probiotic (*n* = 10)	Placebo (*n* = 12)	*p*
Age (years)	51.8 ± 14.9	50.2 ± 9.8	0.773
Men (*n*, %)	8, 80	9, 75	0.781
Body mass index (kg/m^2^)	26.4 ± 4.1	25.2 ± 2.9	0.421
Loose stool frequency (%)	72 ± 7.8	76.6 ± 10.7	0.268
FC (mg/kg)	105.2 ± 92.1	79.3 ± 80.4	
* Log-transformed FC	4.3 ± 0.8	3.9 ± 0.8	0.362
Current smoker (*n*, %)	3, 30	3, 25	0.542
Weekly alcohol intake (g/week)	59.2 ± 58.3	127.4 ± 173.0	0.264
Hypertension (*n*, %)	2, 20	5, 41.6	0.472
Dyslipidemia (*n*, %)	1, 10	2, 16.6	0.521

Data are expressed as the mean ± standard deviation or as the number (percentage). * Since the FC value showed a right-skew, an independent *t*-test was performed with the log-transformed FC value. FC—fecal calprotectin.

**Table 2:** Blood chemistry of the subjects at baseline and two months.

	Baseline			Two Months		
	Probiotic (*n* = 10)	Placebo (*n* = 12)	*p*	Probiotic (*n* = 10)	Placebo (*n* = 12)	*p*
White blood cells (×10^3^)	6.5 ± 2.1	6.1 ± 1.4	0.332	6.2 ± 1.8	6.3 ± 1.8	0.465
Hemoglobin (g/dL)	14.9 ± 1.3	15.2 ± 1.2	0.533	14.7 ± 1.3	14.9 ± 0.9	0.589
ALT (IU/L)	26.3 ± 10.3	26.0 ± 13.7	0.967	23.5 ± 6.3	27.1 ± 15.2	0.486
AST (IU/L)	27.1 ± 13.8	28.0 ± 17.3	0.884	27.9 ± 14.8	24.2 ± 7.6	0.465
BUN	14.4 ± 3.9	12.9 ± 4.2	0.404	14.8 ± 3.4	12.5 ± 3.4	0.452
Creatinine	1.1 ± 0.2	1.0 ± 0.1	0.910	1.1 ± 0.3	1.1 ± 0.2	0.902

Data are expressed as the mean ± standard deviation. ALT, alanine aminotransferase; AST aspartate aminotransferase; BUN—blood urea nitrogen.

## Data Availability

The data in this study are not publicly available but can be requested from the corresponding author.
